# The Medical Profession and Income Tax

**Published:** 1888-08-04

**Authors:** 


### THE EDITOR'S LETTER-BOX.

[Our Correspondents are reminded that prolixity is a great bar to publication, and that brevity of style and conciseness of statement greatly facilitate early insertion.]

### THE MEDICAL PROFESSION AND INCOME TAX.

ALLOW us through your columns to call the attention of all medical practitioners holding appointments or assessed under schedule E to a point we have just carried against the surveyor of taxes, as it forms a very valuable precedent. A district medical officer had never claimed any deductions from his assessment on account of expenses incurred in the performance of his duties, not knowing he could do so. The surveyor of taxes, faithful to his instructions, carefully refrained from telling him of it. This year, however, acting on our advice, Dr. F. claimed repayment of tax for the past three years. The surveyor of taxes returned the form, saying it did not apply to his case, but only to clergymen's expenses, and that he could not now claim the deductions, as they ought to have been claimed at the time of the assessment. We did not accept this fiat of the surveyor, and applied to Somerset House, insisting on the case being decided by the district commissioners, and not by the surveyor. The result was that, although the deductions claimed were not allowed to the full amount, they were admitted to a considerable extent, and an important refund was obtained. The strangest part of the case is that the Inland Revenue enclosed with the post office order the very same form to claim deductions next year as the surveyor of taxes said was not applicable to the case. Kindly let us mention that we have made some important improvements in our forms for balance-sheets and three years returns for the profession to present statement of accounts to the commissioners, either to obtain a fair assessment, to appeal against an unjust one, or to obtain a refund at the end of the year, if the profits have not amounted to the sum upon which income tax was paid.

THE INCOME TAX REPAYMENT AGENCY.

25, Colville Terrace, W.

